# DATASUS as a instrument for developing otologic public health policies

**DOI:** 10.1590/S1808-86942011000300016

**Published:** 2015-10-19

**Authors:** Henrique Fernandes de Oliveira, André Luiz Lopes Sampaio, Carlos Augusto Costa Pires de Oliveira

**Affiliations:** 1 MD. ENT. MSc in Medical Sciences - Medical Sciences School - University of Brasilia; 2 MD. ENT. PhD in Health Sciences - University of Brasília, Associate Physician - ENT and Cochlear Implant Department - University of Brasília; 3 MD. ENT. PhD - Otorhinolaryngology and Head and Neck Surgery Department - University of Brasília; University of Brasília Medical School

**Keywords:** ear, morbidity, socioeconomic factors, health public policy

## Abstract

Ear diseases are illnesses which represent a relevant group of morbidity. Otitis media, for instance, still is a public health problem today.

**Objective:**

To correlate hospital morbidity of ear diseases according to data from the Information Technology Department of the Public Health Care System -SUS (DATASUS), through the Hospital Information System (SIH) with the Human Development Index (IDH) from each unit of the federation. To assess the use of this official data in the creation of otologic public health care policies.

**Materials and Methods:**

Brazilian states were classified according to their respective IDH values. The percentage population from each state was calculated in relation to the entire population of the country, as well as the percentage of hospital admission caused by ear diseases (SIH) for each state in relation to their total number. The hospital admissions from each state were correlated with their respective IDH values.

**Results:**

The state of São Paulo, the third largest IDH was responsible for 38.82% of hospital admissions caused by ear diseases, although its population represents 21.64% of the national population. States with lower IDH had lower hospital admission rates for ear diseases.

**Conclusion:**

SIH, a DATASUS tool, even with limitations, can be an instrument used to create public policies concerning ear diseases.

## INTRODUCTION

Ear diseases represent a relevant group of diseases. Otitis media, for instance, is one of the most prevalent disorders, which today still represents a worldwide public health problem^1^. The socioeconomical impact created by this disease is huge. The inflammatory process often times goes beyond the temporal bone limits, creating severe or even fatal complications. Moreover, it may involve functional hearing loss, either reversible or not.

Despite its still high prevalence, the situation used to be worse. The reduction in ear diseases and, especially, its complications have some explanations. Much of this reduction is credited to a better access the population has to medical care, as well as a shorter time interval between initial signs and symptoms and the medical consultation, when compared to the past.

An efficient public policy to improve medical care concerning ear diseases is only possible when one starts from the epidemiological understanding of this group of diseases.

Official morbimortality data in Brazil are made available by DATASUS^2^ - Information Technology Department of the Brazilian Public Health Care System (SUS). Nonetheless, much is still controversial concerning the feasibility of using it as a tool to create public health policies.

The present study aims at correlating the hospital morbidity of ear diseases in the different Brazilian States with the HDI^3^ - Human development Index - from each state of the country. Thus, one can evaluate whether or not the DATASUS available data can be used to create ear care public policies.

## METHODS

DATASUS encompasses the SUS (SIH/SUS) Hospital Information System as one of its elements. The SIH is based on filling out the Hospital Admission Authorization Form (AIH). We used the TabNet software from the DATASUS website to create the Tables, considering the number of hospital admissions caused by diseases of the ear and of the mastoid apophysis (Chapter VIII from the ICD-10) per state. This system has data from January of 2008 through August of 2010^2^, and data was collected from this entire period. We used data regarding the ear-related causes for hospital admission by the SUS in our country.

The HDI is a comparative measure which uses three aspects: wealth, education and average life expectancy. It is a standardized means of assessment and a measure of the well-being of the population. We used the latest HDI value assigned to each Brazilian state as a means to express the socioeconomic conditions of the respective populations^3^.

The population from each State was obtained from the IBGE - The Brazilian Institute of Geography and Statistics^4^- and we calculated the percentage value it represents in the national population.

The states were considered in ascending order in relation to their respective HDIs. We then calculated the percentage values of hospital admission per ear disease in each State in relation to the total number.

Thus, we compared the percentage value of hospital admission regarding each State with the percentage value of its respective population, correlating it with the respective values of the HDI.

## RESULTS

Table 1 expresses the HDI from each Brazilian State, which varies between 0 and 1.0 - an index of higher socioeconomical level.

**Table 1:** HDI for each Brazilian State.

STATE	HDI	STATE	HDI	STATE	HDI
DF	0.874	MT	0.796	SE	0.742
SC	0.840	AM	0.780	BA	0.742
SP	0.833	AP	0.780	RN	0.738
RJ	0.832	RO	0.776	CE	0.723
RS	0.832	GO	0.776	PB	0.718
PR	0.820	PA	0.775	PE	0.718
ES	0.802	TO	0.756	PI	0.703
MS	0.802	AC	0.751	MA	0.683
MG	0.800	RR	0.750	AL	0.677

Table 2 shows the total value and the hospital stay caused by ear disease from each State, calculated based on the data available at the DATASUS.

**Table 2:** Total and percentage value of hospital stays due to ear diseases for each Brazilian state.

STATE	Hospital Admissions	% of Hospital Admissions
SP	18656	38.82
MG	3802	7.91
RS	3486	7.25
PR	3505	7.29
RJ	2800	5.83
PE	2632	5.48
SC	2129	4.43
BA	2042	4.25
DF	1396	2.91
GO	1207	2.51
CE	1095	2.28
PA	1000	2.08
ES	559	1.16
MT	439	0.91
RN	498	1.04
AM	422	0.88
MS	390	0.81
MA	343	0.71
RO	250	0.52
PI	284	0.59
PB	251	0.52
AC	180	0.37
TO	167	0.35
AL	183	0.38
SE	171	0.36
RR	125	0.26
AP	41	0.09
Total	48053	100

Table 3 depicts the population from each State, as well as their percentage within the national population, obtained from the data available at the IBGE.

**Table 3:** Total and percentage value for each state.

STATE	Population	% total Population
SP	39827570	21.64
MG	19273506	10.47
RJ	15420375	8.38
BA	14080654	7.65
RS	10582840	5.75
PR	10284503	5.58
PE	8485386	4.61
CE	8185286	4.44
PA	7065573	3.84
MA	6118995	3.32
SC	5866252	3.18
GO	5647035	3.06
PB	3641395	1.97
ES	3351669	1.82
AM	3221939	1.75
AL	3037103	1.65
PI	3032421	1.64
RN	3013740	1.63
MT	2854642	1.55
DF	2455903	1.33
MS	2265274	1.23
SE	1939426	1.05
RO	1453756	0.79
TO	1243627	0.67
AC	655385	0.35
AP	587311	0.32
RR	395725	0.21
Total	183987291	100

The State of São Paulo, third highest HDI, was responsible for 38.82% of the hospital admissions caused by ear diseases, although the population represented 21.64% of the national population.

The State of Alagoas, with the lowest HDI, was responsible for only 0.38% of the hospital admissions caused by ear diseases, and its population represents 1.65% of the national population.

## DISCUSSION

The etiology and the factors contributing to pathology are fundamental to understand it and, thus, work on its possible treatment.

Numerous studies have been and continue being carried out in an attempt to unveil the impact of socioeconomical status in disease development. Many of these have concluded that a greater disease expression is associated with a lower socioeconomical status^5^, ^6^, ^7^, ^8^.

Despite their high prevalence throughout the world, we have seen a recent reduction in ear diseases prevalence. Much is attributed to improvements in sanitary conditions and better access to medical care and drug therapy, even if it is still insufficient in many places. Otological complications have also been associated with a worse socioeconomical status^9^, ^10^.

An efficient public policy can be created when policy makers are acquainted with the epidemiology associated with a given disease. In Brazil, SUS (Public Health Care System) data is organized by DATASUS, which provides for free access to a broad data base. In it, for instance, we find information on ear and mastoid morbidity as of January of 2008^2^, broken down by state, as depicted on Table 2. Such morbidity is based on SUS hospital admission records, which are created when the attending physician fills out the AIH (Hospital Admission Authorization form), making up one of the information systems which support DATASUS, especially the SIH.

The HDI is a measure created in 1990, and it has been used by the United Nations Program for Development in order to assess the socioeconomical status of countries^3^. It is based on educational levels, life expectancy at birth and per capita income^3^.

When we analyze the percentage levels of hospital admission per otologic disease concerning each state of the country (Table 2) with the respective percentage numbers of the population (Table 3), we see that some states have a higher morbidity when compared to the total population of the country. And the opposite also happens in some cases. And still, when we associated such values with the respective HDIs (Table 1), we noticed a direct positive relation between a better socioeconomical status and less ear disease morbidity.

These observations are in contrast with the literature because better socioeconomical conditions have been directly associated with better values in the health indicators.

Ear morbidity is greatly associated with disease chronification - most of the times a delay in medical care and/or difficult access to treatment. Thus, it is expected that the morbidity caused by ear disease in a given population reflects, in part, its socioeconomical status. Nevertheless, considering the SIH data, the possible relation is exactly the opposite.

The better interpretation of such results must be another one. The otological morbidity measured by SIH actually reflects hospital morbidity. In other words, since it is based on AIH forms, the data obtained from such system depicts only otological patients with diseases requiring hospital stay. They omit and disregard all of those who have ear diseases, whom did not require or it was not possible to have a hospital approach.

Since most ear-related hospital admissions happen because the patients will be submitted to surgical treatment, the results from the present study enable us to draw other conclusions. The state of São Paulo, with 21.64% of the Brazilian population, had 38.82% of the hospital stays, almost double of what was to be expected, should the division be more equal. This says that in the state of São Paulo, the public health care system is able to surgically treat its otologic patients in a higher number when compared to the national average. On the other hand, the state of Alagoas, with 1.65% of the population, had only 0.38% of the hospital admissions.

The results show us that in our states with low HDI, there are lower rates of hospital admission and, therefore, do not offer much surgical treatment to their otologic patients. If we compare this to the epidemiological data provided by the Brazilian Association of Otorhinolaryngology, we see that those are states with lower numbers of ENT physicians^11^.

The importance of this SUS data system is undeniable. Based on its data, one can identify material and personnel problems and needs and, therefore, propose solutions. Nevertheless, we know that errors do exist, especially concerning information gathering. It is widely known that in many places the AIH forms are incorrectly filled out, because the physicians do not know how to do it, or because they do not give importance to public health care policies. The reliability and reproducibility of the data generated by the SUS information system are fundamental for the creation of reliable public health care policies.

## CONCLUSION

According to hospital morbidity data associated with ear diseases (hospital admissions) available at the SIH, from January of 2008 through August of 2010, the number of hospitalizations is higher (in percentage numbers) in states with higher HDI and lower in those with lower HDI. This reflects the need for personnel and material in the poorer states of the country, which offer less surgical treatment to their ear diseased patients. Therefore, even with limitations, the official information generated by this SUS information system can serve as a tool in the creation of ear-related public health care policies.
